# Communicative health literacy with physicians in healthcare services– results of a Hungarian nationwide survey

**DOI:** 10.1186/s12889-025-21590-1

**Published:** 2025-01-30

**Authors:** Frederico Epalanga Albano Israel, Ferenc Vincze, Róza Ádány, Éva Bíró

**Affiliations:** 1 Doctoral School of Health Sciences, University of Debrecen, Debrecen, Hungary; 2 Department of Public Health and Epidemiology, Faculty of Medicine, University of Debrecen, Debrecen, Hungary; 3 Department of Public Health and Epidemiology, Faculty of Medicine, HUN-REN-UD Public Health Research Group, University of Debrecen, Debrecen, Hungary; 4 National Laboratory for Health Security, Center for Epidemiology and Surveillance, Semmelweis University, Budapest, Hungary; 5 Department of Public Health, Semmelweis University, Budapest, Hungary

**Keywords:** Communicative health literacy, Measurement properties, European health literacy population survey 2019–2021

## Abstract

**Background:**

In an efficient and effective healthcare delivery, good communication plays an essential role. The communicative health literacy (COMM-HL) of the patients is an important attribute, but the number of validated COMM-HL assessment tools is low, and they do not cover all aspects of COMM-HL. That’s why a new scale has been developed within an international collaboration. Our aims are to check the measurement properties of the Hungarian version of this COMM-HL questionnaire, to describe the COMM-HL of the Hungarian adult population and to investigate its determinants.

**Methods:**

A total of 1205 adults completed the telephone interview in 2020 as part of the European Health Literacy Population Survey 2019–2021. The questionnaire included items on sociodemographic data, self-perceived health, social support and COMM-HL. The questionnaire’s measurement properties were assessed using Cronbach’s alpha, Spearman-Brown, and item-total correlation coefficients, while the construct validity was investigated with principal component (PCA) and confirmatory factor analysis. The determinants of the COMM-HL were investigated by linear regression.

**Results:**

Both the value of Cronbach’s alpha and the Spearman-Brown correlation were 0.87. The items belonged to one factor and 62.2% of the total variation was explained by this factor based on the PCA. The fit indices indicated that the one-factor structure of the six-item questionnaire exhibited a good fit for the data. The mean score on the COMM-HL scale was 86.8. The easiest task was explaining health concerns while getting enough time in the consultation was rated as the most difficult one. COMM-HL was lower among females, while it was higher among people aged 66–75 years (compared with 18–25 years), those with greater social support and those without financial deprivation.

**Conclusions:**

The questionnaire can be characterized with good internal consistency and the structure of the COMM-HL questionnaire was best explained as a one factor model. In consultations with patients, the effectiveness of communication should be improved, so that patients do not feel that there is not enough time for consultation. At-risk groups with low COMM-HL need special attention during the interactions and the role of social support has to be clarified, too.

## Background

The term health literacy arose in the literature almost five decades ago and has been defined in many ways. In our study, we rely on the definition of Sørensen and her colleagues, the so called integrated model of health literacy (HL) [1]. According to this model, HL implies knowledge, motivation and competences needed to obtain, understand, process and use information relevant to health in order to make judgments and take decisions regarding healthcare, disease prevention and health promotion to reach a good quality of life. HL has different dimensions, of which communicative HL (COMM-HL) is one. It was first described by Nutbeam as the combination of cognitive, literacy and social skills required to actively participate in everyday activities, to extract information, to derive meaning from different forms of communication and to apply information to changing circumstances [2]. This definition has been further expanded based on the integrated model of HL as a part of an international collaboration [3]. As a result of this process, COMM-HL in healthcare means “patients’ communicative and social skills that enable them to actively engage in face-to-face encounters with healthcare professionals, to give and seek information, derive meaning from it, and apply this information in decision making and in co-producing their health care” [4].

From a public health point of view, it is of utmost importance to be able to measure concepts adequately. That’s why under the umbrella of the Health Literacy Population Survey Project 2019–2021 (HLS_19_) [5] a new tool (HLS19-COM-P questionnaire) has been developed to assess all aspects of COMM-HL according to the previously mentioned definition [4]. HLS_19_ was the first project of the World Health Organization (WHO) Action Network on Measuring Population and Organizational Health Literacy (M-POHL) with 17 participating countries in the WHO European Region, including Hungary.

Several studies on communication have found evidence that the quality of the communication between patients and the healthcare providers plays an essential role in health care, can influence the satisfaction with care [6, 7], and can lead to better outcomes [7, 8, 9]. The HL of the patient is also important, because those with lower levels of HL appear to participate less actively in the medical encounter which could lead to lower health-related knowledge [10, 11, 12] and they trust physicians less [13] and health information from specialist doctors and dentists less [14].

Among the factors associated with COMM-HL, the education level is significantly associated with almost all domains of COMM-HL measured by the Health Literacy Questionnaire, so higher educational level was associated with active engagement with healthcare providers and the ability to find good health information [15]. In addition, the same study found that participants with better financial status were more likely to find good health information; while living in urban areas was significantly associated with active engagement with healthcare providers [15].

Despite the fact that COMM-HL is a prerequisite for better health outcomes and healthcare utilization, to our knowledge there was no complex questionnaire to measure it before the HLS_19_ study and we did not find any Hungarian COMM-HL tool. In our study, we aimed to assess the measurement properties of the Hungarian version of the COMM-HL questionnaire, to describe the COMM-HL of the Hungarian adult population and to determine the factors influencing the COMM-HL.

## Methods

### Study design and sampling

The survey was part of the international Health Literacy Population Survey 2019–2021 [5]. The data collection was carried out on a probability sample (*n* = 1205), selected in a multistage proportional stratification (described in detail elsewhere: [16]) of the Hungarian adult population in 2020. The sample was representative of the Hungarian adult population living in private households in terms of age, sex, educational level and settlement type. Due to the COVID-19 pandemic, computer-assisted telephone interviewing (CATI) was used for data collection. The study was approved by the Medical Research Council Scientific and Research Committee, Hungary (IV/10181-3/2020/EKU). In accordance with the Declaration of Helsinki, informed consent was obtained from all participants.

### Data collection domains and tools

#### Sociodemographic data

We collected information on respondents’ age (categorised as 18–25, 26–35, 36–45, 46–55, 56–65, 66–75, 76+), number of children, sex (male/female), whether they were trained in a health profession (yes/no), and the level of education (highest primary education– maximum primary school–, lower secondary education– vocational school–, secondary education– high school–, tertiary education– college or university degree). We measured self-perceived level of social status on a 1–10 scale, where 1 corresponds the lowest and 10 the highest level in the society. The respondent had to choose the number that represented the step he or she would place himself or herself. A score below 5 can be considered as low social status [5]. Following the international HLS_19_ methodology, the questions on the difficulty of (1) affording needed medication, (2) medical examinations or treatments and (3) paying all bills, were used to assess the respondents’ financial deprivation. The answers were recorded on a four point Likert-scale from very difficult to very easy. The composite measure was calculated as the percentage (measured on a scale of 0-100) of items with valid responses that were answered as “very difficult” or “difficult”, with scores above 50 being considered deprived [5].

#### Self-perceived health and social support

Self-perceived health was measured using a 5 point Likert-scale (1-„very bad”, 5-„very good”), based on the question of the European Health Interview Survey 2019 [17, 18]. The 3-item Oslo Social Support scale was used to measure perceived social support, with responses categorised as poor (total score less than 8 points), moderate (9–11 points) and strong social support (more than 12 points) [17, 18].

#### Communicative health literacy

In order to assess the level of COMM-HL, the Hungarian version of the HLS19-COM-P questionnaire was used. The English version was developed by a working group of the HLS_19_ Consortium. We used the short version (HLS19-COM-P-Q6), where the following six items were measured: “how easy to explain your health concerns to your doctor?”, “how easy to get enough time in the consultation with your doctor?”, “how easy to express your personal views and preferences to your doctor?”, “how easy to ask your doctor questions in the consultation?”, “how easy to be involved in decisions about your health in dialogue with your doctor?”, “how easy to recall the information you get from your doctor?”. The questions could be answered on a four-point Likert scale from “very easy” to “very difficult”. Based on the international recommendation [19], the final COMM-HL score is calculated as the percentage (0-100) of valid items answered with “very easy” or “easy”. If more than 20% of the items contained invalid responses, the score was set to “missing”. A higher score indicates a higher level of COMM-HL.

### Statistical analyses

Firstly we described the sociodemographic characteristics and COMM-HL of the respondents using statistically weighted frequencies with the corresponding 95% confidence intervals (95%CI). This ensured that the estimates reflected the general adult Hungarian population in terms of sex, age, educational level and type of settlement. We used the terminology from the COnsensus-based Standards for the selection of health status Measurement INstruments (COSMIN) taxonomy when described the measurement properties of the questionnaire [20]. Reliability was assessed based on the internal consistency and alternative test-retest analysis. The internal consistency of the COMM-HL scale was measured using Cronbach’s alpha and “alpha if item deleted” (change in coefficient alpha after single component removal), where the value between 0.70 and 0.95 was considered as acceptable reliability [21]. As it was not possible to carry out a “test-retest” analysis with repeated data collection due to the COVID-19 pandemic, an alternative reliability indicator was quantified using the Spearman-Brown split-half reliability method. During the process, the items were randomly assigned into two groups, then the total score on each half of the instrument was computed. These two sets of scores were correlated and then the corrected Spearman-Brown correlation coefficients were quantified. The consistency considered as medium if the correlation was 0.50–0.69, strong with correlations 0.70–0.89, and very strong if the Spearman-Brown coefficient was > 0.90. The Item-Total correlations were also analysed, where a value greater than 0.3 was considered acceptable. As a sensitivity analysis, we also tested the internal consistency of the COMM-HL in the socioeconomic subsamples.

The construct validity was investigated with principal component and confirmatory factor analysis. The factor structure of the questionnaire was established by conducting a principal component analysis (PCA) of valid responses. Kaiser-Meyer-Olkin (KMO) measure of sampling adequacy and Bartlett-test were used to assess the factorability of the data. Spearman’s correlations between items of the questionnaire were calculated, where higher coefficient, suggested stronger correlation between items. Confirmatory factor analysis (CFA) was used to determine whether the items in our instrument support the one-factor structure. The standard guidelines for reporting goodness-of-fit indices were followed: Bollen-Stine bootstrap p-value, Tucker-Lewis index (TLI), comparative fit index (CFI), goodness-of-fit index (GFI), root-mean-square error of approximation (RMSEA), and the p-value of close fit (PCLOSE) were used to assess the model fit. The Bollen-Stine bootstrap p-value (*p* > 0.05) tests the null hypothesis that the model is correct. For TLI, CFI, and GFI, values closer to 1 are better, with values above 0.95 indicating a good fit. Regarding RMSEA, a value below 0.05 indicates a “close fit”. PCLOSE is used to test the close fit of the model, where a p-value greater than 0.05 indicates a close-fitting model [22, 23, 24]. Considering the multivariate non-normality, a bias-corrected (percentile method) bootstrapping procedure (1,000 bootstraps) was used to estimate model parameters.

The relationship between COMM-HL (used in the analyses as a continuous variable) and the sociodemographic, social support and health status variables was investigated by multiple linear regression analyses adjusted for social status, number of children, and presence of health professional training. Associations were quantified using regression coefficients (β) and corresponding 95% confidence intervals (95%CI). The data analysis was carried out by the Stata/IC 16 statistical software’s survey data analysis module and by the SPSS 26.0 software (IBM Corporation, Armonk, New York, USA). The confirmatory factor analysis was conducted using the Amos software, version 29.0.

## Results

### Characteristics of the sample

The representative sampling procedure resulted in 1205 respondents in the study. The weighted distribution of the studied parameters can be seen in Table 1. Around 30% of respondents were aged 35 and under, and the majority of respondents (72.4%) reported that their perceived social status was normal. Predominance of females was seen in the sample, while the lower secondary and secondary educated respondent frequency was almost 67%. Approximately 78% of the respondents was not trained in a health profession, and 70% of the sample had children. Almost 65% of the respondents were in the non-deprived category. The self-perceived health status was assessed as good or very good by 55% (95%CI 52.3–57.9) of the population and 30% (95%CI 27.2–32.5) reported having strong social support.

**Table 1:** Descriptive sample characteristics, weighted by age, sex, education and settlement type

		Weighted proportion (%) (95% CI)
Sex	Male	46.8% (43.9-49.6%)
	Female	53.2% (50.4-56.1%)
Age groups	18–25 years	10.9% (9.3-12.9%)
	26–35 years	19.0% (16.9-21.4%)
	36–45 years	16.3% (14.3-18.5%)
	46–55 years	18.6% (16.5-20.9%)
	56–65 years	15.4% (13.4-17.5%)
	66–75 years	12.9% (11.1-14.9%)
	76 + years	6.8% (5.5-8.4%)
Educational level	Highest primary education	17.6% (15.5-19.8%)
	Lower secondary education	33.3% (30.7-36.1%)
	Secondary education	33.3% (30.7-36.1%)
	Tertiary education	15.8% (13.8-17.9%)
Financial deprivation	Deprived	34.9% (32.1-37.7%)
	Non-deprived	65.1% (62.3-67.9%)
Childrearing	Yes	69.8% (67.2-72.4%)
	No	30.2% (27.6-32.8%)
Training in a health profession	Yes	22.2% (19.9-24.7%)
	No	77.8% (75.3-80.1%)
Self-perceived health	Bad or very bad	8.2% (6.8-9.9%)
	Fair	36.8% (34.0-39.5%)
	Good or very good	55.1% (52.3-57.9%)
Social support	Poor	17.4% (15.3-19.7%)
	Moderate	52.9% (49.9-55.7%)
	Strong	29.8% (27.2-32.5%)
Perceived social status	Low (score below 5)	27.6% (25.1-30.2%)
	Normal (score above 4)	72.4% (69.8-74.9%)

95% CI: 95% confidence interval

### Measurement properties of the questionnaire

#### Reliability testing

The internal consistency measured by the Cronbach’s alpha was 0.87, while the split-half reliability calculated with the Spearman-Brown correlation coefficient was 0.87 for the total sample. The calculated values for the different subsamples can be seen in Table 2. The Cronbach’s alpha for the subsample showed a very narrow range from 0.84 (perceived social status score below 5) to 0.89 (age 76 + years and trained in a health profession). The values of the Spearman-Brown correlation coefficient varied between 0.83 (age 26–35 years) and 0.90 (trained in a health profession).

**Table 2:** Cronbach’s alpha values and Spearman-Brown correlation coefficients for the subsamples

	Cronbach’s alpha	Spearman-Brown coefficient
**Total sample**	**0.87**	**0.87**
Males	0.88	0.88
Females	0.87	0.86
Age 18–25 years	0.86	0.90
Age 26–35 years	0.87	0.83
Age 36–45 years	0.88	0.87
Age 46–55 years	0.87	0.86
Age 56–65 years	0.86	0.88
Age 66–75 years	0.87	0.88
Age 76 + years	0.89	0.88
Highest primary education	0.85	0.86
Lower secondary education	0.87	0.88
Secondary education	0.88	0.84
Tertiary education	0.87	0.87
Perceived social status score below 5	0.84	0.84
Perceived social status score above 4	0.88	0.87
Financial deprivation	0.85	0.86
No financial deprivation	0.87	0.86
Having children	0.87	0.86
No children	0.88	0.89
Trained in a health profession	0.89	0.90
Not trained in a health profession	0.86	0.85

We tested the Cronbach’s coefficient by removing individual items and found that, with the exception of the last item, removing any item results in a decrease in the coefficient. If the last item is removed, the coefficient increases slightly from 0.87 to 0.88. (Table 3) The item-total correlation coefficients were higher than the minimum acceptable level (0.3) in all cases (Table 3). The lowest was for the last item (0.65), while the highest was for the third item (0.85).

**Table 3:** Analysis of the correlation between the items and the whole scale

Items of the scale	Cronbach’s alpha after deleting this item	Item-total correlation coefficient
Item 1: explain your health concerns to your doctor	0.85	0.78
Item 2: get enough time in the consultation with your doctor	0.86	0.78
Item 3: express your personal views and preferences to your doctor	0.83	0.85
Item 4: ask your doctor questions in the consultation	0.84	0.83
Item 5: be involved in decisions about your health in dialogue with your doctor	0.84	0.81
Item 6: recall the information you get from your doctor	0.88	0.65

#### Explanatory factor analyses

Based on the results of the principal components analysis the items belong to one factor (Bartlett-test < 0.001; Kaiser-Meyer-Olkin value: 0.891), 62.2% of the total variation is explained by this one factor (Table 4).

**Table 4:** Result of the principal components analysis

Items of the scale	Factors	Uniqueness
Item 1: explain your health concerns to your doctor	0.79	0.38
Item 2: get enough time in the consultation with your doctor	0.77	0.40
Item 3: express your personal views and preferences to your doctor	0.85	0.27
Item 4: ask your doctor questions in the consultation	0.84	0.29
Item 5: be involved in decisions about your health in dialogue with your doctor	0.82	0.32
Item 6: recall the information you get from your doctor	0.63	0.60
Variance	3.73	
Variance proportion (%)	62.2	

#### Confirmatory factor analysis

**Fig. 1:**
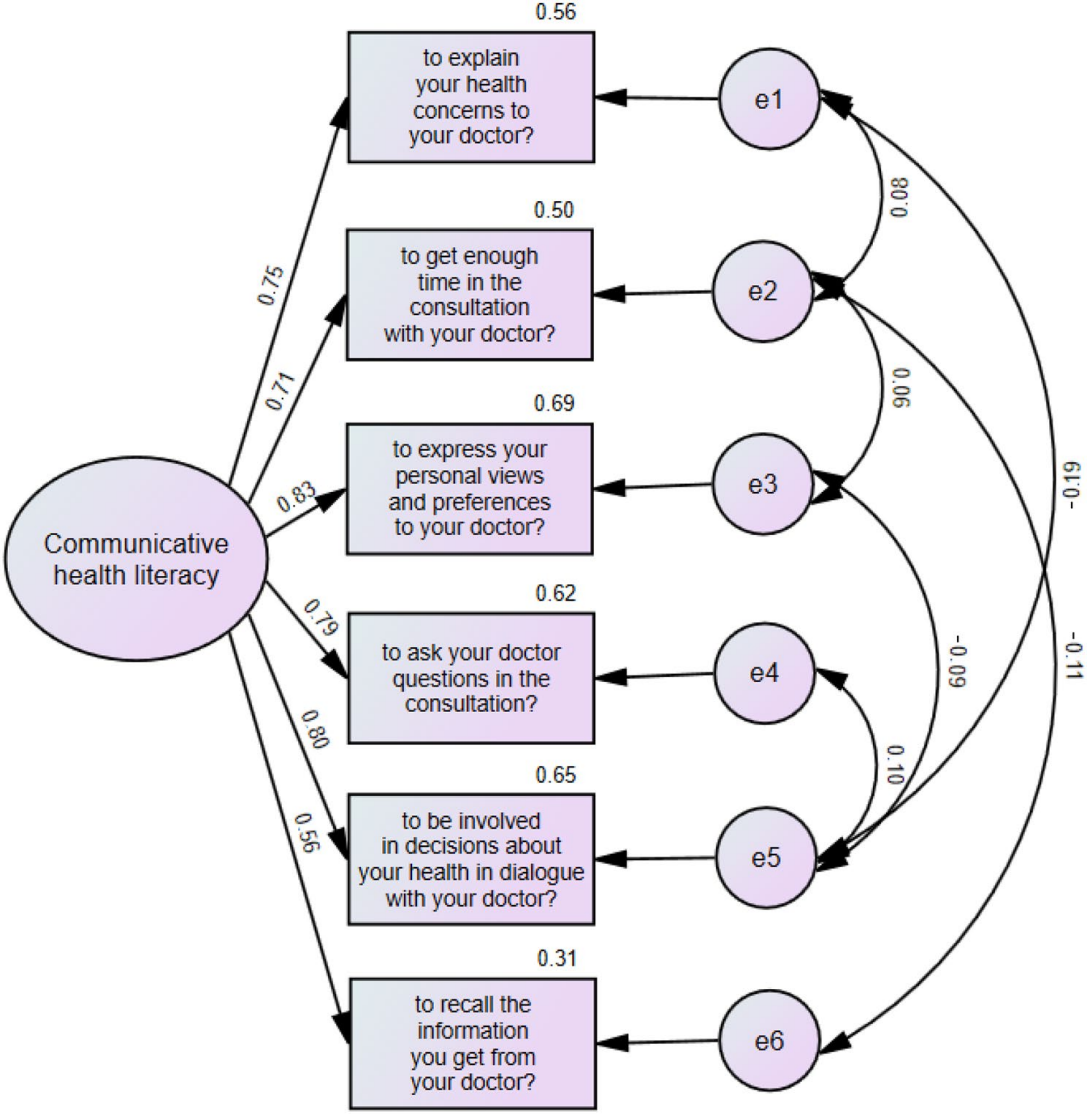
One factor confirmatory factor analysis model of the communicative health literacy questionnaire. Numbers are standardized path coefficients from bootstrapped samples. Overall fit statistics of the model: Bollen-Stine bootstrap p-value: 0.126; TLI - Tucker-Lewis index: 0.988; CFI -comparative fit index: 0.998; GFI - goodness of fit index: 0.997; RMSEA - root mean square error of approximation: 0.047; PCLOSE - p-value of close fit: 0.504

Results of the confirmatory analysis for the model shown in Fig. 1. The fit indices indicated that the one-factor structure of the six-item questionnaire exhibited a good fit for the data. The RMSEA was 0.047 (PCLOSE = 0.504), GFI, TLI, CFI were all above 0.98. As shown in Fig. 1, the standardized regression coefficients for this model ranged from 0.56 to 0.83. All of these coefficients were statistically significant (*p* < 0.01). Figure 1 also shows the squared standardized factor loading, which ranged from 0.313 (Item 6: to recall the information you get from your doctor?) to 0.693 (Item 3: to express your personal views and preferences to your doctor?). COMM-HL accounted for 56% of the variance in question “explain your health concerns to your doctor”, and for 50%, 69%, 62% and 65% of the variance for items “get enough time in the consultation with your doctor”, “express your personal views and preferences to your doctor”, “ask your doctor questions in the consultation” and “involved in decisions about your health in dialogue with your doctor”, respectively. The lowest true score variance was measured in the item of “recall the information you get from your doctor”, where only 31% of the variance was reliable variance. The correlation coefficients ranged from 0.35 to 0.68 among the item scores. The Spearman’s bivariate correlation coefficients were the lowest for the item “to recall the information you get from your doctor?” (Table 5).

**Table 5:** Spearman’s bivariate correlations between the items of the questionnaire on communicative health literacy

	Item 1	Item 2	Item 3	Item 4	Item 5	Item 6
Item 1: to explain your health concerns to your doctor?	-					
Item 2: to get enough time in the consultation with your doctor?	0.57 (*p* < 0.001)	-				
Item 3: to express your personal views and preferences to your doctor?	0.64 (*p* < 0.001)	0.63 (*p* < 0.001)	-			
Item 4: to ask your doctor questions in the consultation?	0.60 (*p* < 0.001)	0.58 (*p* < 0.001)	0.67 (*p* < 0.001)	-		
Item 5: to be involved in decisions about your health in dialogue with your doctor?	0.53 (*p* < 0.001)	0.56 (*p* < 0.001)	0.64 (*p* < 0.001)	0.68 (*p* < 0.001)	-	
Item 6: to recall the information you get from your doctor?	0.41 (*p* < 0.001)	0.35 (*p* < 0.001)	0.47 (*p* < 0.001)	0.48 (*p* < 0.001)	0.50 (*p* < 0.001)	-
M	3.17	2.95	3.05	3.13	3.04	3.21
SD	0.58	0.69	0.61	0.58	0.61	0.59

M: mean, SD: standard deviation

### The communicative health literacy of the Hungarian adults

**Fig. 2:**
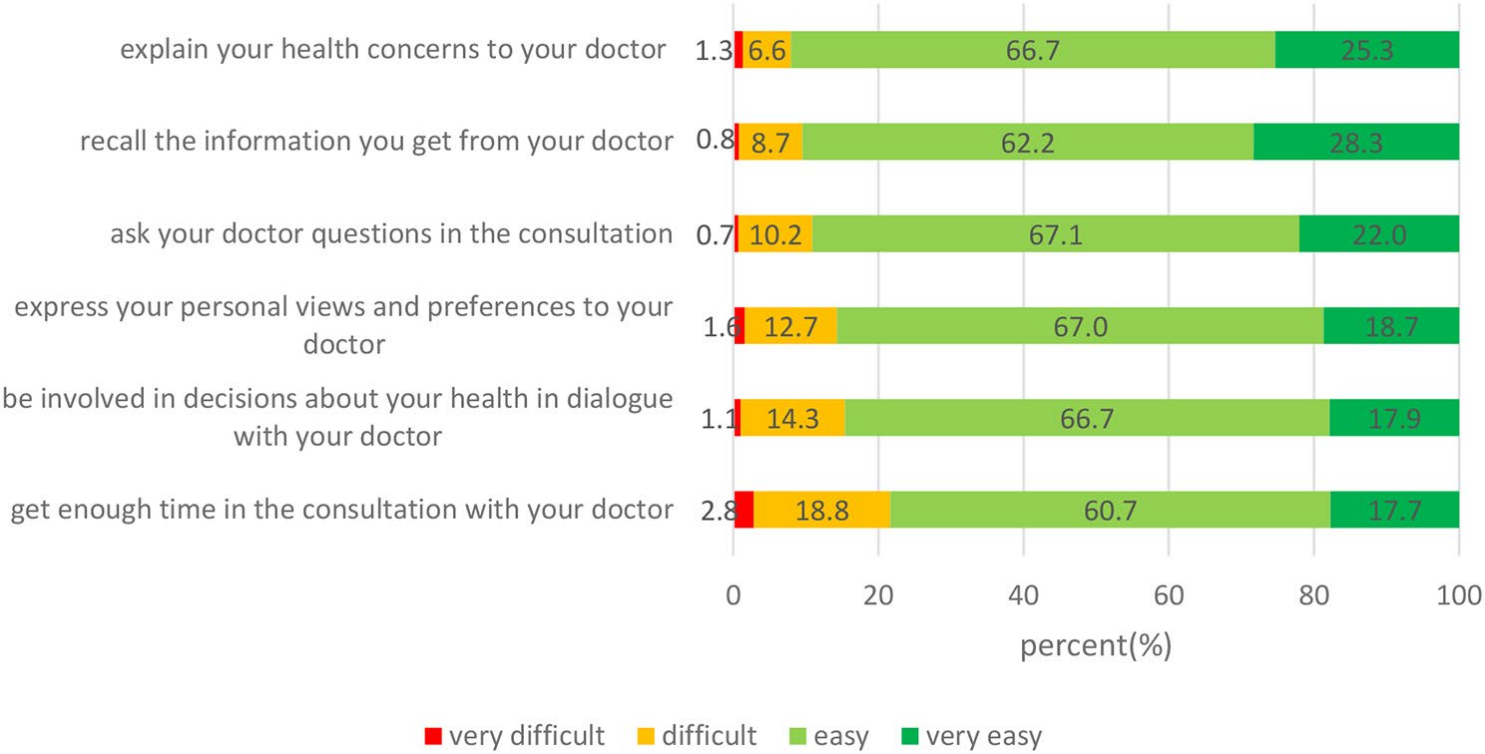
Percentage of answers on each item of the communicative health literacy scale (weighted data*). Note: Introductory question in the English version: “On a scale from very easy to very difficult, how easy would you say it is for you to…” which was also used in the Hungarian version. * Weighted by age, sex, education and settlement type

The weighted mean score of the COMM-HL scale was 86.8 (95%CI 84.8–88.8). The weighted mean score of the COMM-HL scale was the lowest among those suffering from financial deprivation, poor social support and low social status, while the highest scores were among those who receiving strong social support and living without financial deprivation (Table 6). The most difficult item was „to get enough time in the consultation with your doctor” ((very) difficult: 21.6%), followed by “to be involved in decisions about your health in dialogue with your doctor” ((very) difficult: 15.4%). While „to explain your health concerns to your doctor” ((very) difficult: 7.9%) was the easiest item. Figure 2 shows the detailed results.

**Table 6:** The weighted mean of the communicative health literacy scale by the key sample subgroups

		Weighted mean^*^ (95% CI)
Sex	Male	88.8 (85.1–92.4)
	Female	85.1 (81.5–88.7)
Age groups	18–25 years	84.2 (77.7–90.7)
	26–35 years	86.9 (75.1–98.7)
	36–45 years	84.1 (80.5–87.8)
	46–55 years	89.8 (87.5–92.2)
	56–65 years	86.7 (82.7–90.6)
	66–75 years	89.6 (82.1–97.2)
	76 + years	84.1 (76.8–91.4)
Educational level	Highest primary education	84.6 (83.4–85.9)
	Lower secondary education	89.1 (86.3–91.9)
	Secondary education	89.6 (79.8–99.4)
	Tertiary education	88.6 (80.6–96.6)
Financial deprivation	Deprived	79.2 (74.8–83.6)
	Non-deprived	90.7 (89.1–92.4)
Childrearing	Yes	86.6 (76.7–96.6)
	No	86.9 (84.8–89.1)
Training in a health profession	Yes	88.5 (79.7–97.3)
	No	86.3 (86.1–86.5)
Self-perceived health	Bad or very bad	82.8 (69.0-96.7)
	Fair	85.1 (78.8–91.4)
	Good or very good	88.8 (86.4–90.8)
Social support	Poor	79.5 (72.1–86.9)
	Moderate	86.2 (8.20–89.3)
	Strong	92.0 (86.4–97.6)
Perceived social status	Low (score below 5)	80.9 (76.5–85.2)
	Normal (score above 4)	89.0 (85.3–92.7)

* Weighted by age, sex, education and settlement type. 95% CI: 95% confidence interval

### The determinants of communicative health literacy

In comparison to the respondents within the 18–25 age, the level of COMM-HL was increased amongst those within the 66–75 age group (β: 8.39, p: 0.013), in the case of the lack of financial deprivation (β: 6.90, *p* < 0.001) and strong social support (β: 8.00, *p* < 0.001), while it was lower among females (β: -2.89, p: 0.036). There was no association between COMM-HL and education, self-perceived health, social status, childrearing, health professional training (Table 7).

**Table 7:** The determinants of communicative health literacy according to the multivariate linear regression analyses

Dependent variables	β* (95% CI)	*p*-value
Sex (ref: male)	**-2.89 (-5.58;-0.19)**	**0.036**
Age groups (ref: 18–25 years)		
26–35 years	3.63 (-2.82;10.08)	0.270
36–45 years	2.31 (-4.36;8.98)	0.498
46–55 years	5.93 (-0.67;12.52)	0.078
56–65 years	5.46 (-1.23;12.15)	0.109
66–75 years	**8.39 (1.77;15.01)**	**0.013**
76 + years	5.29 (-2.19;12.77)	0.165
Education (ref: highest primary education)		
Lower secondary education	-0.13 (-4.54;4.27)	0.952
Secondary education	3.00 (-1.27;7.28)	0.168
Tertiary education	-1.90 (-6.76;2.95)	0.442
Lack of financial deprivation (ref: deprivation)	**6.90 (3.71;10.08)**	**< 0.001**
Self-perceived health (ref: bad/very bad)		
Fair	0.01 (-5.00;5.01)	0.998
Good/very good	1.55 (-3.70;6.81)	0.563
Social support (ref: poor)		
Moderate	3.05 (-0.74;6.85)	0.115
Strong	**8.00 (3.83;12.17)**	**< 0.001**

β (95% CI): linear regression coefficient (95% confidence interval), ref: reference category

*Adjusted for social status, childrearing, and training in health profession

Significant results are highlighted in bold

## Discussion

This work emphasises the efforts to measure the psychometric properties of the Hungarian version of a tool for assessing COMM-HL with physicians in health services, which can be used easily and not time consuming in healthcare settings. The instrument used in this research helps to understand the real communicative challenges faced by healthcare users, especially during the consultations with physicians. Our secondary objectives were to describe the COMM-HL of the Hungarian adults using the HLS19-COM-P-Q6 instrument and to assess its determinants.

The good reliability coefficient indicates that the two halves of the test are highly correlated and that the questionnaire has good internal consistency. These findings can be interpreted as each item in the test measuring the same property as the whole test. Based on our analysis, no change in the item set is needed to improve the reliability and consistency of the scale. The results of the factor analyses suggest that a one factor structure is appropriate for the COMM-HL model, with the six questions measuring the same construct. Using CFA, it was found that the structure of the communicative health literacy questionnaire was best explained as a one factor model, and this configuration seems to be in line with our original theoretical framework. Except one item (“recall the information you get from your doctor”), the relatively high standardized factor loadings suggested good reliability of the indicators which means the indicators highly correlated with the latent factor communicative health literacy. The tool showed favourable measurement properties on a large, representative sample and could be used to assess the COMM-HL level of Hungarian adults.

The mean score of the COMM-HL scale was 86.8, which is significantly higher than the international average score (83.2 points) of the HLS_19_ study [4]. More than one-fifth of respondents had difficulties with getting enough time in consultations with their doctor– similar to other six of nine countries where this scale was used in the HLS_19_ study–, and almost one in seven had difficulties being involved in decisions about their health in dialogue with their doctor. Explaining health concerns to their doctor was the easiest task, both nationally and internationally [4].

Based on our results, the effectiveness of communication should be improved, so that patients do not feel that there is not enough time for consultation with the physician. The effectiveness of communication could be enhanced by improving the communication skills of health professionals (e.g. using images/pictures as illustration where it is possible, using simple and clear language), including the teaching of techniques like “teach back” [25] or “chunk-and-check” [26]. On the other hand, communication between the health care users should be facilitated. For example, the employment of patient educators or mediators who may help to improve communication in consultations can be useful [27], or the introduction of “Ask Me 3” patient educational program which encourages patients to ask their doctor what their main problem is, what they need to do to get better, and why it is important for them to do so [28].

In our study, COMM-HL was increased with better financial status and strong social support, while it was lower among females. In the HLS_19_ study, a similar trend was found for sex and financial status, while the direction of the association between COMM-HL, age and education was inconsistent across the countries [4].

In terms of sex, an Ethiopian COMM-HL study among patients [15] found that being female was not associated with most domains of the COMM-HL, except lower odds of actively managing health, while higher education level and income lead to a higher odds of finding health information, but they used a different COMM-HL scale. In another study with a different COMM-HL tool, no age, sex or self-perceived health status difference was found relating to the COMM-HL score, but higher education level was associated with a higher score [29]. In a Dutch study of patients with chronic diseases, lower levels of COMM-HL were found for patients with higher age, lower education and lower income [30]. There were no significant age-, sex- and education-related differences in COMM-HL scores, but scores were higher for those with higher self-rated economic status among Japanese diabetic patients [31]. Our results are only consistent with previous studies regarding the positive association between financial status and COMM-HL and no association between self-reported health status and COMM-HL, but comparability is limited given the methodological differences between published studies.

For better policy and decision making, tailoring the real determinants of communicative health literacy is vital for healthcare improvement, as a good implementation program depends on the rational decision taken prior to the implementation. During patient-physician interactions, at-risk groups with low COMM-HL need special attention [32], partly by focusing on the use of the above mentioned techniques. Another option could be the introduction of interventions (e.g. community programs or involving caregivers of low HL persons) where the positive effect of social support could be beneficial to improve COMM-HL [33].

### Strengths and limitations

It should be acknowledged that there are limitations to the present study. Firstly, because the results and conclusions were drawn from a cross-sectional study design, so we cannot make any judgments about causal relationships. Secondly, the study used computer-assisted telephone interviews to collect data. However, it should be noted that the use of different data collection techniques, such as computer-assisted personal interviewing or computer-assisted web interviewing, could affect the results. To investigate this, further studies are needed. Also, the focus of the instrument was limited to the COMM-HL in relation to physician-patient interaction and not to health professionals in general. Furthermore, it is hard to assess the source of the difficulties measured, as perceived communication difficulties may be also related to characteristics of the physicians/health care system. Previous Hungarian studies have used patient-reported experiences [34] and shared decision-making [35–36] to obtain information about these aspects. However, only the patient side was investigated in the present study.

As a strength, the study consistently followed the international guideline during the planning, nationwide sampling, and data collection, which is likely to have reduced the risk of any systematic errors that may have occurred.

## Conclusion

In conclusion, the questionnaire can be characterized with good internal consistency, so it could be used to assess the COMM-HL in the Hungarian population. Regarding the potential solutions to the patients’ difficulties, the effectiveness of the communication should be improved involving the physicians and the health care system, which could help to avoid the feeling that there is not enough time for the consultation. Risk groups with low COMM-HL need special attention during the interactions and the potential role of social support in improving COMM-HL has to be clarified.

## Data Availability

The datasets used and/or analysed during the current study are available from the corresponding author on reasonable request.

## Abbreviations

β: Linear regression coefficients
CATI: Computer-assisted telephone interviewing
CFA: Confirmatory factor analysis
CFI: Comparative fit index
95%CI: 95% confidence intervals
COMM-HL: Communicative health literacy
COSMIN: COnsensus-based Standards for the selection of health status Measurement INstruments
COVID-19: Coronavirus disease 2019
GFI: Goodness-of-fit index
HL: Health literacy
HLS19: Health Literacy Population Survey Project 2019–2021
HLS19-COM-P: Communicative health literacy questionnaire
HLS19-COM-P-Q6: Short version of the communicative health literacy questionnaire
IBM: International Business Machines
KMO: Kaiser-Meyer-Olkin
M-POHL: World Health Organization Action Network on Measuring Population and Organizational Health Literacy
PCA: Principal component analysis
PCLOSE: p-value of close fit
SD: Standard deviation
RMSEA: Root-mean-square error of approximation
TLI: Tucker-Lewis index
USA: United States of America
WHO: World Health Organization

## References

[1] Sørensen K, Van den Broucke S, Fullam J, et al. Health literacy and public health: a systematic review and integration of definitions and models. BMC Public Health. 2012;12:80. 10.1186/1471-2458-12-80. 22276600 10.1186/1471-2458-12-80 PMC3292515

[2] Nutbeam D. Health literacy as a public health goal: a challenge for contemporary health education and communication strategies into the 21st century. Health Promot Int. 2000;15(3):259–67.

[3] Finbråten HS, Nowak P, Griebler R, Bíró É, Vrdelja M, Charafeddine R, Griese L, Bøggild H, Schaeffer D, Link T, et al. The HLS19-COM-P, a New Instrument for Measuring Communicative Health Literacy in Interaction with Physicians: Development and Validation in nine European countries. Int J Environ Res Public Health. 2022;19:11592. 10.3390/ijerph191811592. 36141865 10.3390/ijerph191811592 PMC9517091

[4] Nowak P, Finbråten HS, Biro E, Bøggild H, Charafeddine R, Mancini J, Griebler R, Griese L, Kucera Z, Link T, et al. Communicative Health Literacy with physicians in health care services. The HLS19 Consortium of the WHO Action Network M-POHL: International Report on the methodology, results, and recommendations of the European Health Literacy Population Survey 2019–2021 (HLS19) of M-POHL. Vienna: Austrian National Public Health Institute; 2021. pp. 233–74.

[5] The HLS19 Consortium of the WHO Action Network M-POHL. International Report on the methodology, results, and recommendations of the European Health Literacy Population Survey 2019–2021 (HLS19) of M-POHL. Vienna: Austrian National Public Health Institute; 2021.

[6] Clever SL, Jin L, Levinson W, Meltzer DO. Does doctor-patient communication affect patient satisfaction with hospital care? Results of an analysis with a novel instrumental variable. Health Serv Res. 2008;43(5 Pt 1):1505–19. 10.1111/j.1475-6773.2008.00849.x. 18459954 10.1111/j.1475-6773.2008.00849.x PMC2653895

[7] Sharkiya SH. Quality communication can improve patient-centred health outcomes among older patients: a rapid review. BMC Health Serv Res. 2023;23:886. 10.1186/s12913-023-09869-8. 37608376 10.1186/s12913-023-09869-8 PMC10464255

[8] Street RL Jr, Makoul G, Arora NK, Epstein RM. How does communication heal? Pathways linking clinician-patient communication to health outcomes. Patient Educ Couns. 2009;74(3):295–301. 10.1016/j.pec.2008.11.015. 10.1016/j.pec.2008.11.015 19150199

[9] Tavakoly Sany SB, Behzhad F, Ferns G, Peyman N. Communication skills training for physicians improves health literacy and medical outcomes among patients with hypertension: a randomized controlled trial. BMC Health Serv Res. 2020;20(1):60. 10.1186/s12913-020-4901-8. 31973765 10.1186/s12913-020-4901-8 PMC6979365

[10] Katz MG, Jacobson TA, Veledar E, Kripalani S. Patient literacy and question-asking behavior during the medical encounter: a mixed-methods analysis. J Gen Intern Med. 2007;22(6):782–6. 10.1007/s11606-007-0184-6. 17431697 10.1007/s11606-007-0184-6 PMC2583801

[11] Paasche-Orlow MK, Wolf MS. The causal pathways linking health literacy to health outcomes. Am J Health Behav. 2007;31(Suppl 1):19–26. 10.5555/ajhb.2007.31.supp.S19. 10.5555/ajhb.2007.31.supp.S19 17931132

[12] Schillinger D, Bindman A, Wang F, Stewart A, Piette J. Functional health literacy and the quality of physician-patient communication among diabetes patients. Patient Educ Couns. 2004;52(3):315–23. 10.1016/S0738-3991(03)00107-1. 14998602 10.1016/S0738-3991(03)00107-1

[13] Oguro N, Yajima N, Miyawaki Y, Yoshimi R, Shimojima Y, Sada KE, Hayashi K, Shidahara K, Sakurai N, Hidekawa C, Kishida D, Ichikawa T, Ishikawa Y, Kurita N. Effect of communicative and Critical Health Literacy on Trust in Physicians among patients with systemic Lupus Erythematosus (SLE): the TRUMP2-SLE project. J Rheumatol. 2023;50(5):649–55. 10.3899/jrheum.220678. 36379567 10.3899/jrheum.220678

[14] Chen X, Hay JL, Waters EA, Kiviniemi MT, Biddle C, Schofield E, Li Y, Kaphingst K, Orom H. Health Literacy and Use and trust in Health Information. J Health Commun. 2018;23(8):724–34. 10.1080/10810730.2018.1511658. 30160641 10.1080/10810730.2018.1511658 PMC6295319

[15] Tilahun D, Abera A, Nemera G. Communicative health literacy in patients with non-communicable diseases in Ethiopia: a cross-sectional study. Trop Med Health. 2021;49:57. 10.1186/s41182-021-00345-9. 34256862 10.1186/s41182-021-00345-9 PMC8276450

[16] Bíró É, Vincze F, Nagy-Pénzes G, Ádány R. Investigation of the relationship of general and digital health literacy with various health-related outcomes. Front Public Health. 2023;11:1229734. 10.3389/fpubh.2023.1229734. 37588120 10.3389/fpubh.2023.1229734 PMC10426797

[17] Központi Statisztikai Hivatal. Európai Lakossági Egészségfelmérés 2019 kérdőív [European Health Interview Survey of 2019– Hungarian version Questionnaire]. Budapest: Központi Statisztikai Hivatal. 2019. https://www.ksh.hu/elef/elef2019_kerdoiv.pdf Accessed 06 Apr 2024.

[18] Eurostat. European health interview survey (EHIS wave 3) methodological manual re-edition 2020. Luxemburg: European Commission, Publications Office of the European Union. 2020. https://ec.europa.eu/eurostat/documents/3859598/10820524/KS-01-20-253-EN-N.pdf/2d66d5d7-b966-38ba-881a-a8f4b6d3f5e0?t=1588680461000 Accessed 06 Apr 2024.

[19] The HLS19 Consortium of the WHO Action Network M-POHL. The HLS19-COM-P instrument to measure communicative health literacy. Factsheet. Vienna: Austrian National Public Health Institute; 2022.

[20] Mokkink LB, Terwee CB, Patrick DL, Alonso J, Stratford PW, Knol DL, Bouter LM, de Vet HC. The COSMIN study reached international consensus on taxonomy, terminology, and definitions of measurement properties for health-related patient-reported outcomes. J Clin Epidemiol. 2010;63(7):737–45. 10.1016/j.jclinepi.2010.02.006. 20494804 10.1016/j.jclinepi.2010.02.006

[21] Terwee CB, Bot SD, de Boer MR, van der Windt DA, Knol DL, Dekker J, Bouter LM, de Vet HC. Quality criteria were proposed for measurement properties of health status questionnaires. J Clin Epidemiol. 2007;60(1):34–42. 10.1016/j.jclinepi.2006.03.012. 17161752 10.1016/j.jclinepi.2006.03.012

[22] Kline RB. Principles and practice of structural equation modeling. 3rd ed. Guilford Press: New York, NY, USA;; 2011.

[23] Hu L, Bentler PM. Cutoff criteria for fit indexes in Covariance structure analysis: conventional criteria versus New Alternatives. Struct Equ Model Multidiscip J. 1999;6(1):1–55. 10.1080/10705519909540118.

[24] Byrne BM. Structural equation modelling with AMOS: basic concepts, applications and programming. NJ: Lawrence Erlbaum Associates, Inc.; 2001.

[25] Agency for Healthcare Research and Quality Use the Teach-Back Method. Tool 5. Content last reviewed February 2024, Rockville, MD. https://www.ahrq.gov/health-literacy/improve/precautions/tool5.html Accessed 06 Apr 2024.

[26] Canberra Health Literacy, Chunk. and Check. 2020. https://cbrhl.org.au/health-services-providers/communicating-with-consumers/chunk-and-check/ Accessed 06 Apr 2024.

[27] Kósa K, Katona C, Papp M, Sándor J, Fürjes G, Bíró K, Ádány R. Segéd-egészségőrök működése az Alapellátás-fejlesztési Modellprogram praxisközösségeiben [The role and activity of Health mediators in the Primary Health Care Model Programme of praxis communities]. Egészségtudomány. 2021;65(2):39–50. 10.29179/EgTud.2021.2.39-50.

[28] Institute for Healthcare Improvement. Ask Me 3: Good Questions for Your Good Health https://www.ihi.org/resources/tools/ask-me-3-good-questions-your-good-health Accessed 06 Apr 2024.

[29] Chinn D, McCarthy C. All aspects of health literacy scale (AAHLS): developing a tool to measure functional, communicative and critical health literacy in primary healthcare settings. Patient Educ Couns. 2013;90(2):247–53. 10.1016/j.pec.2012.10.019. 23206659 10.1016/j.pec.2012.10.019

[30] Heijmans M, Waverijn G, Rademakers J, van der Vaart R, Rijken M. Functional, communicative and critical health literacy of chronic disease patients and their importance for self-management. Patient Educ Couns. 2015;98(1):41–8. 10.1016/j.pec.2014.10.006. 25455794 10.1016/j.pec.2014.10.006

[31] Ishikawa H, Takeuchi T, Yano E. Measuring functional, communicative, and critical health literacy among diabetic patients. Diabetes Care. 2008;31(5):874–9. 10.2337/dc07-1932. 18299446 10.2337/dc07-1932

[32] Pérez-Stable EJ, El-Toukhy S. Communicating with diverse patients: how patient and clinician factors affect disparities. Patient Educ Couns. 2018;101(12):2186–94. 10.1016/j.pec.2018.08.021. 30146407 10.1016/j.pec.2018.08.021 PMC6417094

[33] Lee SY, Arozullah AM, Cho YI. Health literacy, social support, and health: a research agenda. Soc Sci Med. 2004;58(7):1309–21. 10.1016/S0277-9536(03)00329-0. 14759678 10.1016/S0277-9536(03)00329-0

[34] Zrubka Z, Brito Fernandes Ó, Baji P, Hajdu O, Kovacs L, Kringos D, Klazinga N, Gulácsi L, Brodszky V, Rencz F, Péntek M. Exploring eHealth literacy and patient-reported experiences with Outpatient Care in the Hungarian General Adult Population: cross-sectional study. J Med Internet Res. 2020;22(8):e19013. 10.2196/19013. 32667891 10.2196/19013 PMC7448194

[35] Brito Fernandes Ó, Hölgyesi Á, Péntek M. Patient-centred care in Hungary: contributions to foster a policy agenda. Z Evid Fortbild Qual Gesundhwes. 2022;171:58–61. 10.1016/j.zefq.2022.04.015. 35618623 10.1016/j.zefq.2022.04.015

[36] Rencz F, Tamási B, Brodszky V, Gulácsi L, Weszl M, Péntek M. Validity and reliability of the 9-item Shared decision making questionnaire (SDM-Q-9) in a national survey in Hungary. Eur J Health Econ. 2019;20(Suppl 1):43–55. 10.1007/s10198-019-01061-2. 31111402 10.1007/s10198-019-01061-2 PMC6544590

